# In situ synchrotron diffraction and modeling of non-equilibrium solidification of a MnFeCoNiCu alloy

**DOI:** 10.1038/s41598-021-85430-z

**Published:** 2021-03-15

**Authors:** Benjamin Schneiderman, Andrew Chihpin Chuang, Peter Kenesei, Zhenzhen Yu

**Affiliations:** 1 Colorado School of Mines, Golden, CO 80401 USA; 2 Advanced Photon Source, Argonne National Laboratory, Lemont, IL 60439 USA

**Keywords:** Materials science, Structural materials, Techniques and instrumentation, Theory and computation

## Abstract

The solidification mechanism and segregation behavior of laser-melted Mn_35_Fe_5_Co_20_Ni_20_Cu_20_ was firstly investigated via in situ synchrotron x-ray diffraction at millisecond temporal resolution. The transient composition evolution of the random solid solution during sequential solidification of dendritic and interdendritic regions complicates the analysis of synchrotron diffraction data via any single conventional tool, such as Rietveld refinement. Therefore, a novel approach combining a hard-sphere approximation model, thermodynamic simulation, thermal expansion measurement and microstructural characterization was developed to assist in a fundamental understanding of the evolution of local composition, lattice parameter, and dendrite volume fraction corresponding to the diffraction data. This methodology yields self-consistent results across different methods. Via this approach, four distinct stages were identified, including: (I) FCC dendrite solidification, (II) solidification of FCC interdendritic region, (III) solid-state interdiffusion and (IV) final cooling with marginal diffusion. It was found out that in Stage I, Cu and Mn were rejected into liquid as Mn_35_Fe_5_Co_20_Ni_20_Cu_20_ solidified dendritically. During Stage II, the lattice parameter disparity between dendrite and interdendritic region escalated as Cu and Mn continued segregating into the interdendritic region. After complete solidification, during Stage III, the lattice parameter disparity gradually decreases, demonstrating a degree of composition homogenization. The volume fraction of dendrites slightly grew from 58.3 to 65.5%, based on the evolving composition profile across a dendrite/interdendritic interface in diffusion calculations. Postmortem metallography further confirmed that dendrites have a volume fraction of 64.7% ± 5.3% in the final microstructure.

## Introduction

Multi principal component alloys (MPCAs), often called high-entropy alloys (HEAs), are an emerging class of materials that have immense versatility in their design space. Early studies developing the theory behind these alloys reasoned that systems containing five or more principal alloying elements would tend to exhibit single solid solution phases, which are thermodynamically stabilized by their high configurational entropy^1^. The widely investigated equimolar CrMnFeCoNi MPCA developed by Cantor et al. was considered as a proof-of-concept, as it possesses a homogenous single-phase face-centered cubic (FCC) structure over a wide temperature range^2^. More recently, Otto et al. challenged this assumption by selectively replacing one of the elements in Cantor’s system to fabricate several other MPCAs. None of these equimolar variant alloys displayed single-phase behavior except for Cantor’s alloy^3^. This finding indicates that element selection is critical in MPCA design. Along with reports that many of the desirable properties of MPCAs (e.g., a combination of excellent strength and ductility) are relevant even in more complex microstructures^4^, it also sparked a groundbreaking new research avenue exploring the design of multi-phase and segregated MPCAs that are of engineering value^5^. Though this avenue is less than a decade in the making, in certain MPCA systems, segregations have already been suggested to augment strength (at the expense of ductility) in both numerical^6^ and experimental^7^ investigations.

In the authors’ previous study^8^, a MnFeCoNiCu MPCA with the approximate composition of Mn_35_Fe_5_Co_20_Ni_20_Cu_20_ was demonstrated to be a good candidate brazing filler for repair of Ni-base superalloys. This composition was selected because its liquidus temperature is substantially below the melting range of the base metal, Ni-base Alloy 600, and its narrow solidification range improves its viability for defect-free brazing^8^. The as-cast microstructure of Mn_35_Fe_5_Co_20_Ni_20_Cu_20_ consists of Fe- and Co-rich dendrites and Mn- and Cu-rich interdendritic regions. However, in laboratory-scale x-ray diffraction (XRD) experiments, a single set of FCC diffraction peaks was observed, and the local variations in composition can be eliminated through a homogenization heat treatment at 950 °C. These two findings demonstrate that the direct solidification substructure in the as-cast material represents a non-equilibrium condition, rather than a two-phase equilibrium. Braze joints of Alloy 600 with the MPCA filler displayed centerline Cu–Mn–Ni segregations, indicating a similar non-equilibrium compositional heterogeneity.

Several studies have proposed Cu to be responsible for compositional segregation in as-solidified MPCAs, due to its positive binary enthalpy of mixing with other common constituent elements^3,7,9–15^. The mechanism for such segregation behavior, however, remains unclear, as there are limited accounts of in situ research in MPCA solidification. For MPCAs in general, a handful of studies^11,16,17^ have employed in situ XRD to observe solid-state phase transformations under pressure and external stress in MPCAs, but studies that discuss the direct solidification behavior of MPCAs, especially the segregation behavior, are largely limited to postmortem analyses^14,15,18–25^. Some of these ex situ investigations^15,22,26^ on segregation behavior of Cu-containing MPCAs propose the mechanism of liquid-phase separation to be in play, with the evidence of Cu-rich globular clusters present within the solidified microstructures. Another study^24^ discusses the rejection of Cu as a solute during solidification, due to its lower affinity for other MPCA elements. This paper aims to examine in situ the solidification and segregation behavior of Mn_35_Fe_5_Co_20_Ni_20_Cu_20_ MPCA, which provides critical insights for facilitating its application as an engineering alloy.

To this end, in situ synchrotron XRD was performed during an autogenous laser welding process to monitor the evolution of segregation during and after solidification of the MPCA, at millisecond temporal resolution. For conventional alloys systems where well defined crystallographic structures exist, Rietveld method^27^ is a commonly used and proven analytical tool to provide the relative amounts of each microconstituent present at every stage in the real-time diffraction data. Many software packages, such as GSAS-II^28^, have been developed for Rietveld refinement. However, in monitoring the dendritic solidification of an MPCA, the following three challenges arise in performing a traditional data analysis: (1) The dendritic and interdendritic microconstituents represent the same crystallographic structure with a slightly different composition. Diffraction peaks corresponding to each will therefore display a significant overlap. No disparity in crystal structure exists by which to quantitatively assign peaks to specific microconstituents; (2) For MPCAs studied in present work, the constituent elements have nearly indistinguishable x-ray scattering factor; (3) no single input crystal structure is representative of a random, disordered, quinary solid solution, without including a computationally prohibitive number of atomic configurations in the structure. To overcome these challenges, a novel approach combining a hard-sphere approximation model, thermodynamic simulation, thermal expansion measurement and microstructural characterization was developed to enable in-depth analysis of the evolution of local composition, lattice parameter, and ultimately dendrite volume fraction corresponding to the solidification and subsequent solid-state diffusion.

## Methods

### Specimen fabrication

Samples for laser-melting were cut from an as-cast Mn_35_Fe_5_Co_20_Ni_20_Cu_20_ MPCA button that was arc-melted under argon from its pure constituent elements, a process described with more details in^8^. To maximize compositional accuracy during arc-melting, compensatory Mn was added to the button to make up for Mn that was lost to vaporization or Mn pieces breaking up violently upon contact with the arc. After ensuring the appropriate mass of Mn had been added to the alloy, the button was flipped and re-melted three times to maximize bulk compositional homogeneity. The samples that were cut from this button for in situ laser welding were ground using SiC abrasive paper until all visible oxide on the surface was removed, and subsequently cold-rolled to a final thickness of approximately 500 µm. Specimens 30 mm long and 3 mm wide were then cut from this foil for laser welding. Dilatometry specimens were also prepared directly from an as-cast MPCA button by milling the material to rectangular dimensions of 9 mm × 3 mm × 3 mm using a 3/8-in carbide end mill.

### Laser melting and In situ XRD

**Figure 1:**
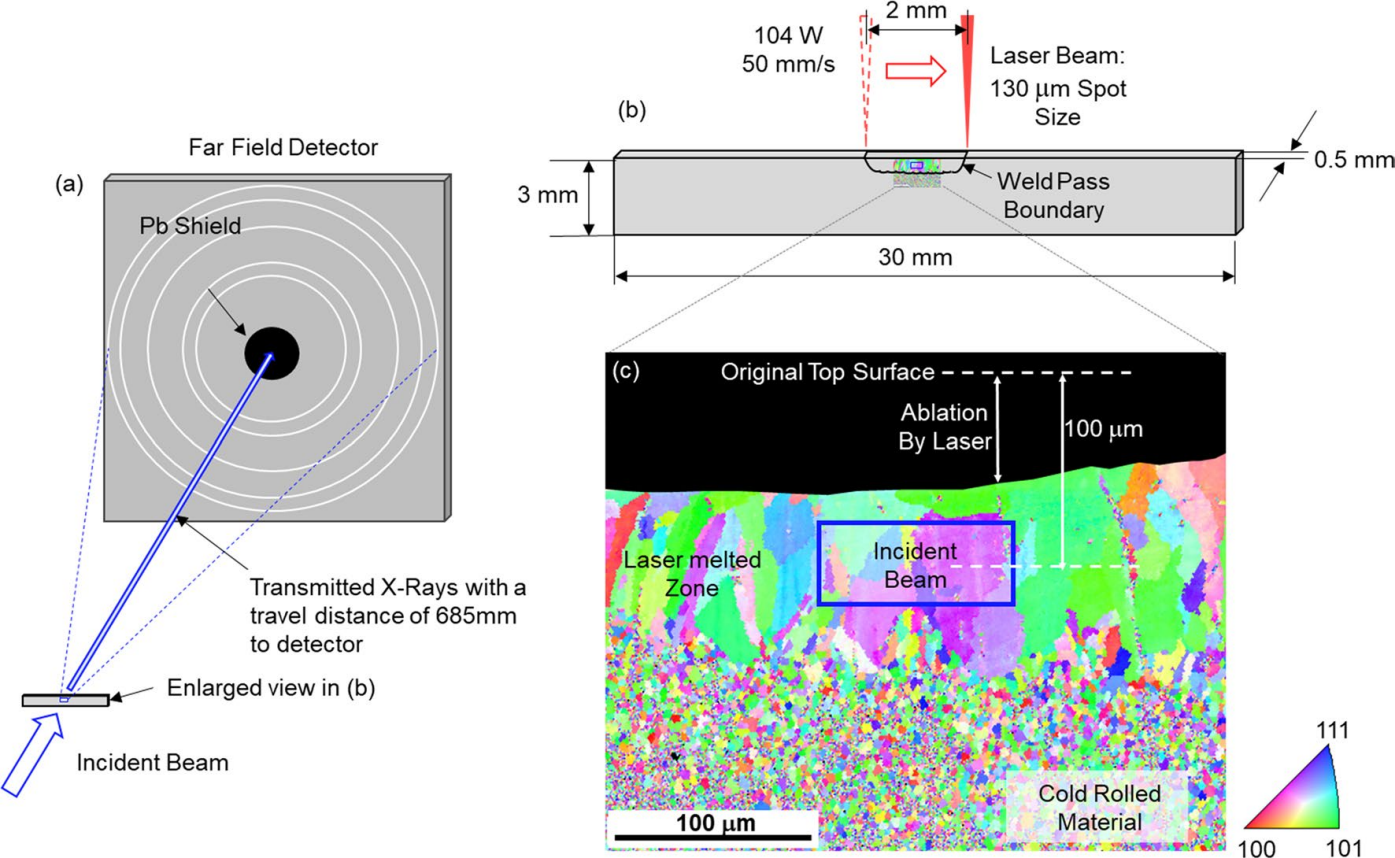
(**a**) In situ x-ray diffraction experiment setup. (**b**) Specimen geometry and laser melting setup (not to scale). (**c**) EBSD inverse pole figure showing grain morphology on the front face of the specimen after laser melting. The size and position of the beam are shown to scale.

In situ synchrotron-generated x-ray diffraction experiments were conducted at the Advanced Photon Source (APS) beamline 1-ID-E. A full schematic of the experiment setup is shown in Fig. 1. A monochromatic x-ray beam with an energy of 55.62 keV was directed through the thickness of the MPCA samples. Simultaneously, a ytterbium fiber laser (IPG model YLR-500-AC-Y11) with a wavelength of 1070 nm was scanning across the top edge of the sample and melting the uppermost portion, to generate an autogenous weld pass, as shown in Fig. 1b. The laser parameters used for melting the samples were held constant at a power of 104 W, a travel velocity of 50 mm/s, and a laser spot size of 130 µm at the sample surface. During the experiments, the samples were held inside a vacuum chamber filled with argon gas to minimize oxidation. The approximate penetration of the autogenous laser weld is illustrated by the extent of the large, re-solidified grains visible in the inverse pole figure map in Fig. 1c, as a clear boundary is evident between these and the fine grains within the un-melted cold-rolled foil.

A Pilatus3 X CdTe hybrid photon counting detector was positioned approximately 685 mm downstream to the specimen, and was used to measure the diffraction patterns of the entire melting, solidification and cooling process, as shown in Fig. 1a. The diffraction patterns were collected at a frame rate of 250 Hz (4 ms per frame) with exposure time of 1 ms to capture the rapid evolution of phases during melting and cooling. The data was collected with minimum duration of 3 s for each laser melting event. Static patterns of the specimen with a longer exposure time of 100 ms were also collected before and after the in situ experiments. In all cases, the incident X-ray beam was focused with a pair of compound-refractive lenses (CRL) to a final beam size of 100 µm horizontally (defined by slits) and 40 µm vertically (full width at half maximum of focused beam). The center of the x-ray beam was positioned 100 microns below the top edge of the sample prior to melting, as shown in Fig. 1c. Note that this edge was ablated by laser melting.

### Metallographic and compositional analysis

Metallographic characterization was carried out on laser-melted samples. Samples were mounted in Bakelite, ground with SiC abrasive paper, and polished with a final step of 0.05 micron colloidal silica using standard metallographic preparation procedures. Electron backscatter diffraction (EBSD) and energy dispersive spectroscopy (EDS) data were collected on polished specimens using a JEOL-7000F field-emission scanning electron microscope (SEM) with a working distance of 18 mm. Samples were then etched by swabbing with undiluted aqua regia (3:1 HCl to HNO_3_) for 10–15 s. Etched samples were used for optical imaging and further SEM study for the purpose of estimating dendrite fraction.

### Thermal expansion analysis

The milled rectangular dilatometry specimens underwent heating in a TA Instruments DIL 805L push-rod dilatometer employing quartz rods, to collect data on the coefficient of thermal expansion (CTE) as a function of temperature for the MPCA. Heating was performed under vacuum at a constant rate of 10 °C/s to a final temperature of 950 °C to avoid approaching the MPCA melting temperature. CTE data was extrapolated from 950 °C to the liquidus temperature.

## Experimental results

### Segregation behavior from postmortem characterization

**Figure 2:**
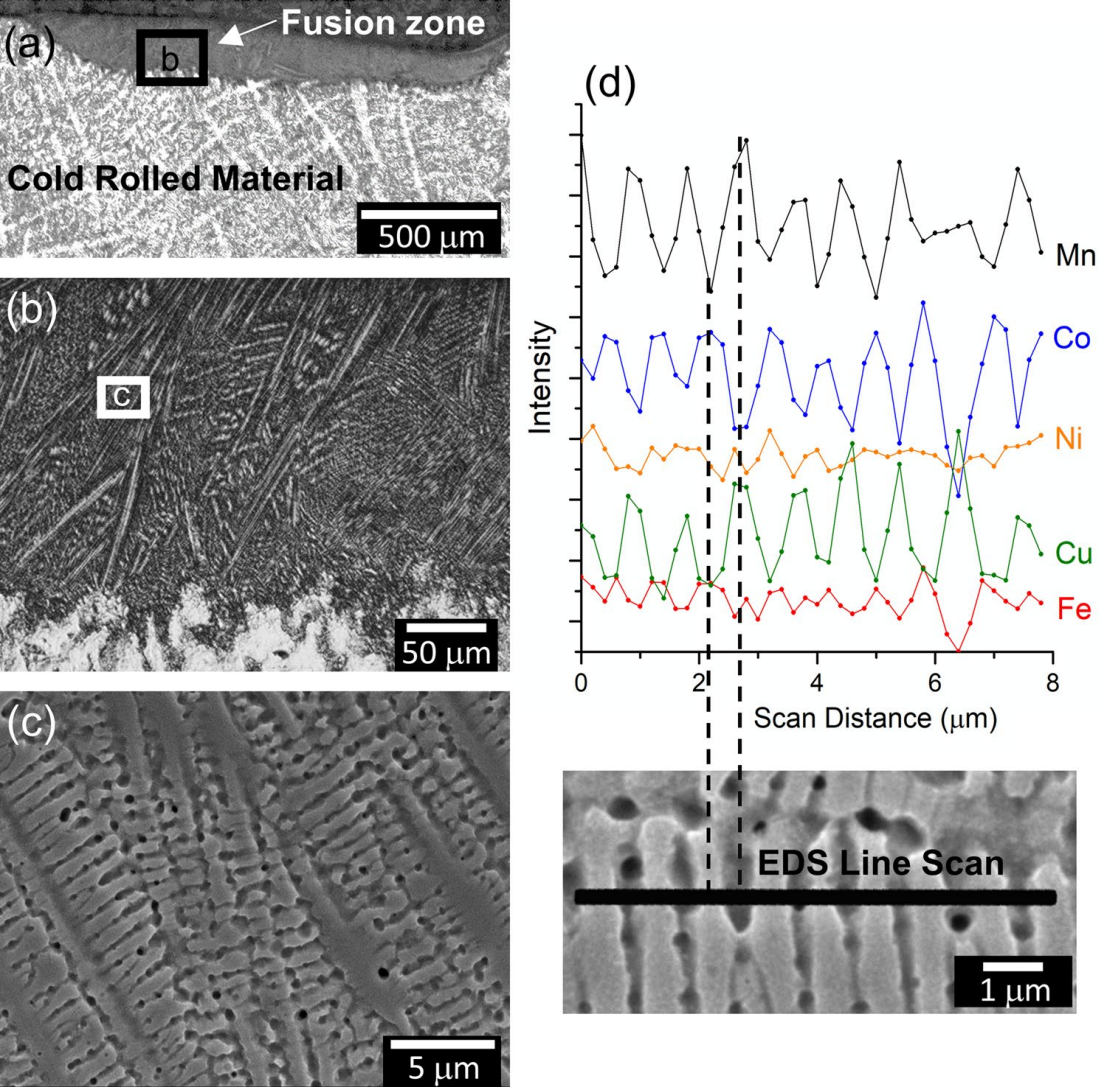
Microstructure of the MPCA specimen after laser-melting. (**a**,**b**) Etched optical micrographs displaying (**a**) the full extent of fusion zone and (**b**) a closer view of the dendritic microstructure; (**c**) Secondary electron image illustrating primary and secondary dendrite arms; and (**d**) EDS line scan result and magnified view of the scan location across a series of secondary dendrite arms.

Figure 2 shows the microstructure of the laser re-melted material. The selected etchant preferentially dissolves interdendritic material, leaving the dendrites in relief on the surface. The low-magnification optical micrograph in Fig. 2a displays the full fusion zone as well as the unaffected cold rolled material. The coarse dendritic microstructure resulting from the original arc-casting process is preserved in the cold rolled material, since no annealing or homogenization treatment was performed prior to cold rolling. The fusion zone possesses a highly refined dendritic microstructure clearly evident in Fig. 2b,c. The high magnification EDS line scan shown in Fig. 2d qualitatively displays compositional segregation between the dendritic (light-colored) and interdendritic (dark-colored) regions. The EDS line scan was performed transversely to a series of secondary dendrite arms and displays oscillatory behavior in the signal from each element. Fe and Co oscillate in phase with one another, displaying segregation to the dendrites, while Mn and Cu are clearly segregating to the interdendritic space. Ni cannot be concluded to segregate to either from this profile. This data qualitatively agrees with the segregation behavior reported for the arc-melted alloy^8^ and most non-equilibrium solidified materials in the MnFeCoNiCu system^7,9–13^. Note that the average secondary dendrite arm spacing ($\lambda$) is measured to be approximately 800 nm for the re-solidified material.

### Analysis of solidification mechanism via in situ XRD

**Figure 3:**
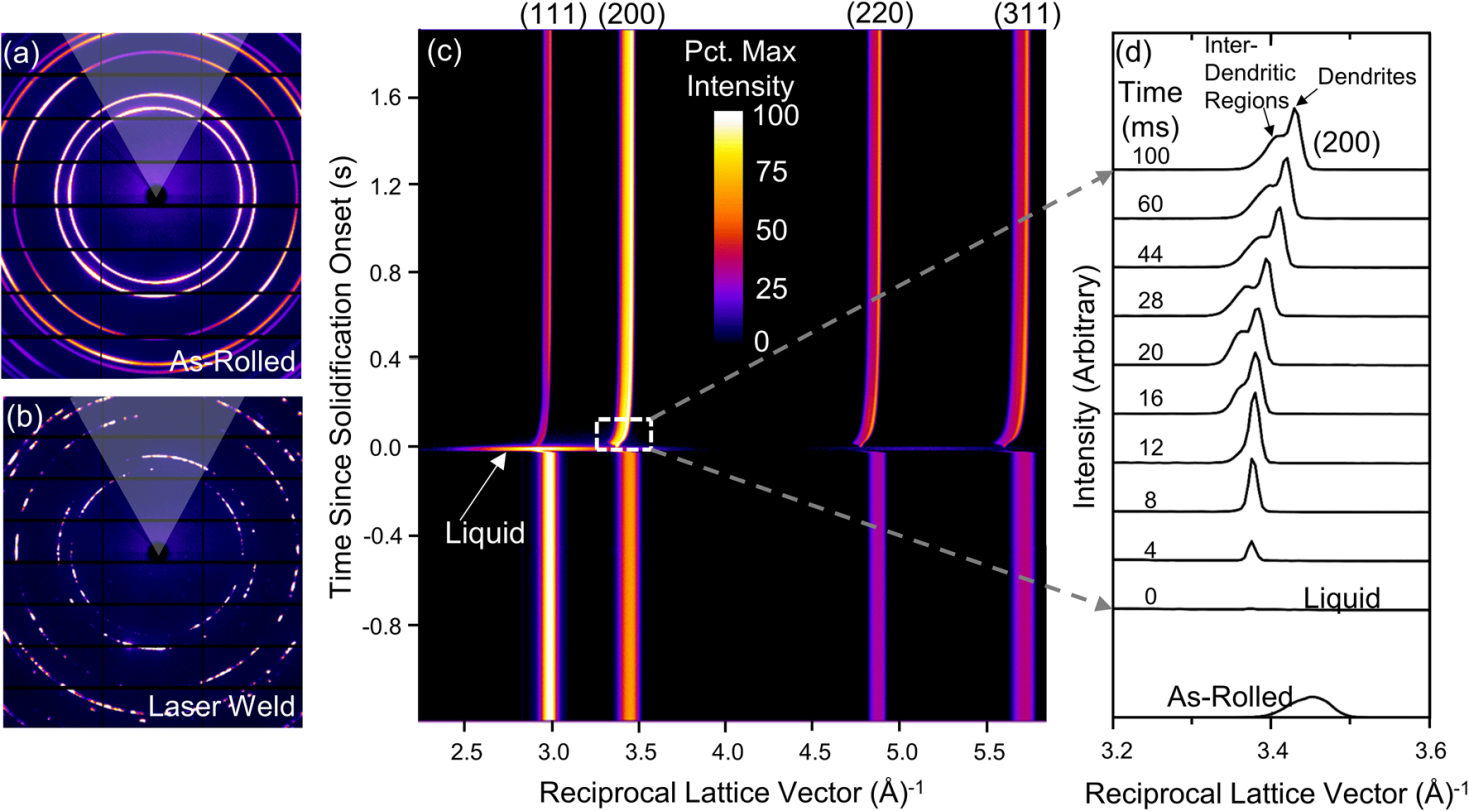
In situ synchrotron x-ray diffraction data: (**a**,**b**) Raw detector images resulting from ex situ 100 ms beam exposures on (**a**) rolled material before the laser scan and (**b**) fully cooled, re-solidified material after the laser scan. White overlays in (**a-b**) represent the azimuthal range for diffraction pattern integration. (**c**) Visual representation of the in situ evolution of XRD patterns before and after the onset of solidification. Brightness corresponds to normalized diffracted intensity as shown by the color scale. (**d**) Integrated (200) diffraction peak at selected time intervals following the onset of re-solidification.

Figure 3 displays the diffraction measurement results. Raw images of the Debye–Scherrer rings collected by the far-field detector before and after laser welding are provided in Fig. 3a,b. Figure 3a shows the 100 ms exposure diffraction pattern produced by the rolled material prior to re-melting. The continuity of the rings in this pattern is a signature of the refined grain size produced from rolling, shown from the EBSD result in Fig. 1c to be on the order of 1–5 µm in average diameter. The fine grain size allowed the incident beam to collect data from a large grain population, which diffracted x-rays in all directions to form continuous rings. However, this is not a perfect powder pattern, as rolling texture is evident in the presence of poles visible as bright locations along several of the rings. Figure 3b shows the 100 ms exposure diffraction pattern after laser melting and complete cooling. Continuity of the rings shown in the 2D pattern is substantially diminished due to increased grain size in the fusion zone (20–50 µm), which is consistent with the EBSD map shown in Fig. 1c. Integration of the two-dimensional (2D) diffraction data was performed over an azimuthal range ± 30° from the vertical, illustrated by the white wedges in Fig. 3 (a-b).

The one-dimensional (1D) in situ diffraction data before and after the onset of solidification is displayed visually over time in Fig. 3c, with the color scale corresponding to normalized diffracted intensity. All 1D integrated data is plotted against the reciprocal lattice vector, $q=4\pi \mathrm{sin}\theta /\lambda$, where $\theta$ is the Bragg angle and $\lambda$ is wavelength in Angstroms. All peak positions can be indexed as displayed to an FCC crystal structure. The full liquid state lasts for a duration of approximately 20 ms, during which the 2D diffraction patterns show a broad amorphous hump without any sharp peaks corresponding to crystalline phases. We defined the last frame that shows a fully amorphous pattern as the reference point (${t}_{0}$) in the time axis for our analysis in this work. Subsequent solidification and cooling results in a rapid evolution of both peak position and morphology for approximately 100 ms, after which the diffraction pattern remains largely unchanging.

As illustrated in the diffraction pattern from fully cooled material in Fig. 3b, the strong (200) intensity in the upward vertical direction (azimuth near 90°) indicates that the primary dendrite arms grew along a [200] direction following the heat flow with maximum temperature gradient. This is consistent with the preferred solidification texture of cubic materials, as noted for the case of austenitic stainless steel welds^29^. The high (200) intensity in the azimuthal integration range made this peak well-suited for further analysis, and Fig. 3d shows the evolution of the integrated (200) peak at selected in situ frames in the transient period during the early stage of solidification. The evolution of the diffraction peak as a function of time provides significant insights into the solidification behavior of this HEA system. For the first $12\;{\text{ ms}}$ after the first crystalline phase grows into the field-of-view at ${t}_{0}$, a sharp diffraction peak emerges from amorphous background. The shape of the (200) peak remains relatively symmetrical, indicating only primary dendrite arms grow into the liquid. By ${t}_{0}+16\;{\text{ ms}}$, a distinct, broad shoulder has emerged on the left side of the peak, indicating a segregated FCC composition with a larger lattice parameter is forming within the interdendritic region. This shoulder reaches its maximum separation from the main peak at approximately ${t}_{0}+28\;{\text{ ms}}$, before its severity is moderated as the sample continues to cool.

**Figure 4:**
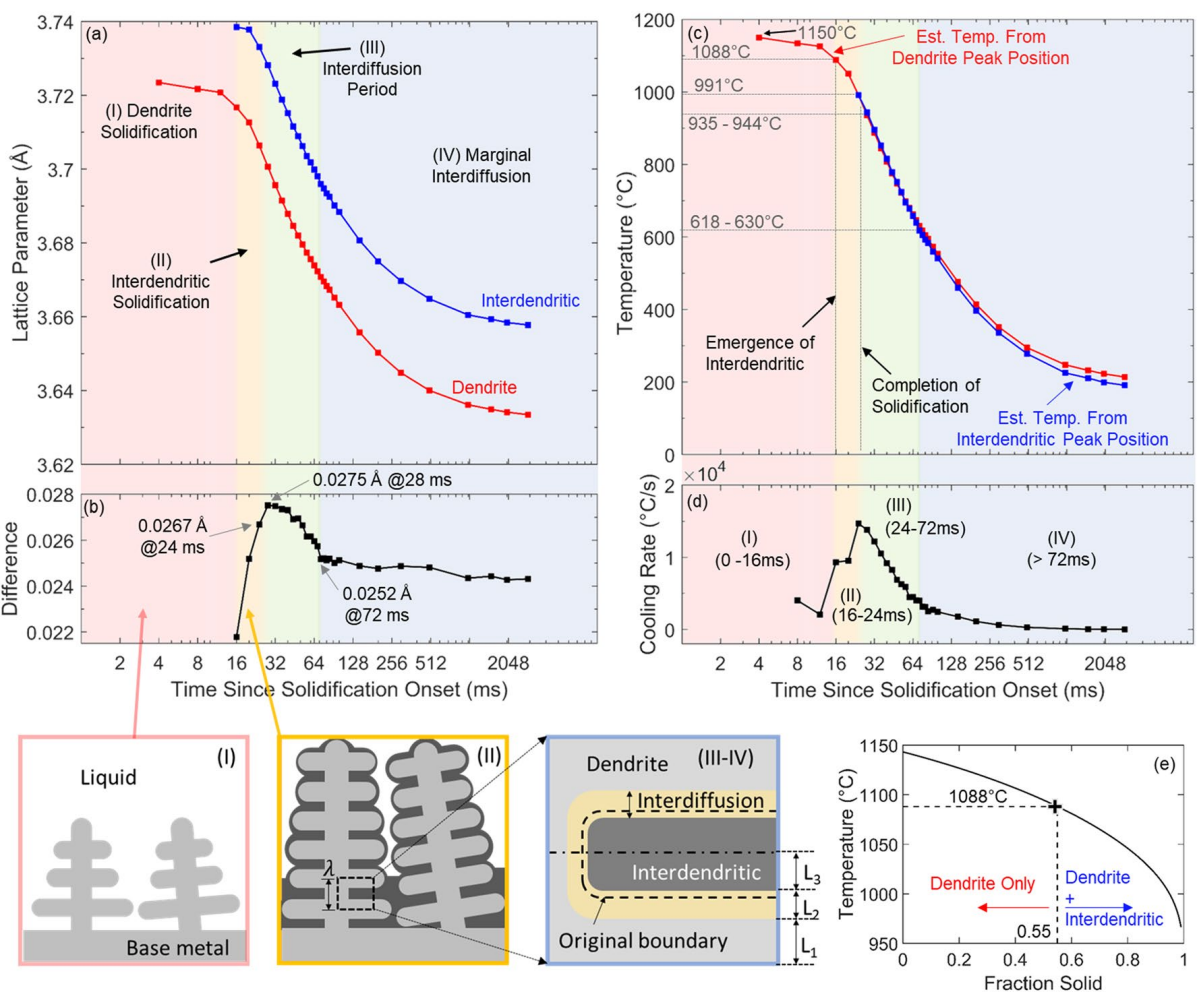
Solidification behavior analysis from experimental (200) XRD data: (**a**) Time-evolution of FCC lattice parameters corresponding to the dendritic and inter-dendritic peaks, determined from Pearson VII functions fit to the data; (**b**) Time-evolution of the lattice parameter difference between the dendritic and inter-dendritic regions; (**c**) Temperature profiles estimated based on lattice parameter evolution and using the variable CTE data collected through dilatometry; (**d**) The instantaneous cooling rate calculated from the temperature profile; and (**e**) Scheil solidification curve indicating the fraction of solidifying material partitioning to the dendritic and inter-dendritic regions, based on the temperature at the termination of Stage I. Schematics illustrate the dendritic microstructure evolution of the MPCA at each stage of solidification and cooling.

The SEM micrographs shown in Fig. 2 and the diffraction peak evolution in Fig. 3d both suggest a staged solidification mechanism, in which dendrites solidify first (represented by the main peak) followed by formation of the inter-dendritic material with a slightly different composition (the shoulder peak). Detailed analysis of the peak evolution yields information about the timing of these stages and the temperature profiles, as summarized in Fig. 4.

**Figure 5:**
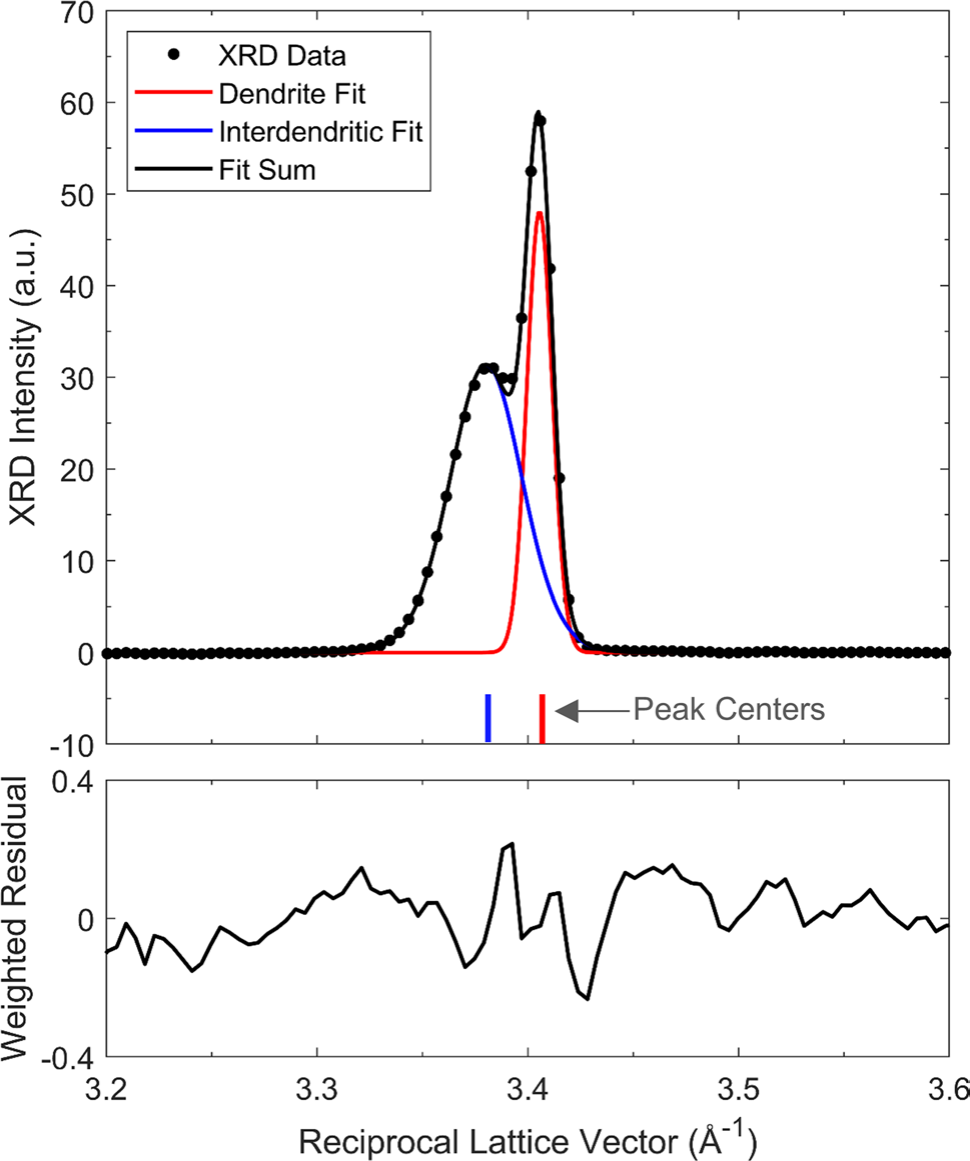
Example of a (200) XRD frame fit using two separate Pearson VII functions. The centers of the fitted peaks were used to calculate the FCC lattice parameters of the dendritic and interdendritic compositions, respectively.

The time-evolution curves of the lattice parameters associated with both (200) FCC peaks, plotted in Fig. 4a, were determined by fitting two separate Pearson VII functions^30^ to the in situ (200) diffraction data. Pearson VII was selected over the pseudo-Voigt function^31^ because the former was more robust in its ability to resolve the shoulder peak from the main peak. The center of each fitted function was used to estimate the lattice parameter, as demonstrated by an example diffraction frame shown in Fig. 5. During the first 12–16 ms following the onset of solidification, only the main peak produces a calculable Pearson VII fit. After the shoulder peak (represented by the blue curve in Fig. 5) emerges, the lattice parameter difference between the two regions evolves, as shown in Fig. 4b. The authors note that using exclusively the (200) peak is not sufficient to accurately determine the lattice parameter. However, the strong solidification texture and large re-solidified grains rendered the intensity of the other diffraction peaks unavailable for reliable peak analysis. The approach employed here is a compromise, under the as-solidified material conditions, to quantify the evolution of lattice spacing as a function of time during solidification.

In the high-thermal-gradient environment imposed by laser melting, undercooling phenomena can be neglected. Hence, it is reasonable to assume that the onset of solidification corresponds with the known MPCA liquidus temperature of 1150 °C, measured through differential thermal analysis^8^. Beginning at this known temperature, it is possible to estimate the temperature at each subsequent diffraction frame by tracking the shift in lattice parameter, via a recursive method represented by Eq. (1).

1 $${T}_{i}= {T}_{i-1}+\left(\frac{{a}_{i}- {a}_{i-1}}{CTE\left({T}_{i-1}\right)*{a}_{i-1}}\right)$$

*T*_*i*_ represents the estimate at a given diffraction frame (*i*), while *T*_*i*−1_ represents the temperature at the previous frame (*i*−1). The subscripts carry the same meaning throughout the equation, and *a* represents the lattice parameter measured through the diffraction data, while *CTE*(*T*_*i*−1_) is the temperature-dependent coefficient of thermal expansion determined from the bulk dilatometry experiment. Applying Eq. (1) recursively for each diffraction frame as time advances produces the estimated temperature profile shown in Fig. 4c. Since the interdendritic peak does not yet exist at the known liquidus temperature of 1150 °C, only the dendrite peak was used in determining the values of *a*_*i*_ and *a*_*i-1*_ in Eq. (1) until the interdendritic peak is fully established at ${t}_{0}+24\;{\text{ ms}}$. Afterward, the temperature is also estimated using the interdendritic peak, assuming the calculated temperature at ${t}_{0}+24\;{\text{ ms}}$ from the dendrite peak is the starting point for the recursive analysis of the interdendritic peak. It was assumed that the bulk data for coefficient of thermal expansion was representative of both the dendrites and the interdendritic regions. Diffusional exchange between the dendrite and interdendritic material causes the peaks to shift toward one another, independently of a temperature-induced shift. This diffusional exchange will be discussed in more details in the section covering thermodynamic simulations. Since cooling causes both peaks to shift rightward, the diffusion-induced rightward shift in the interdendritic peak leads to an over-estimate of cooling from this peak, and the corresponding leftward shift in the dendritic peak leads to an under-estimate of cooling. Hence, the true temperature estimate is bounded by the red and blue curves in Fig. 4c. Numerical calculation of the time-derivative of the temperature profile gives the instantaneous cooling rate shown in Fig. 4d.

Examining the evolutions of lattice parameter difference (Fig. 4b) and cooling rate (Fig. 4d) allows for the definition of four distinct stages, as illustrated by the schematics in Fig. 4. Stage I (0-16 ms) represents dendritic solidification in a liquid pool and terminates at the emergence of the shoulder peak. In Stage II, the inter-dendritic region solidifies and the difference between the dendritic and inter-dendritic lattice parameters continuously increases as the stage progresses. This increase indicates continued solidification of a composition that becomes increasingly disparate from that of the dendrite. The difference in lattice parameter reaches its maximum at $t={t}_{0}+28\;{\text{ ms}}$. The cooling rate in Fig. 4d steadily climbs from late in Stage I and reaches its maximum value of nearly $1.5\times$ 10^4^ °C/s at $t={t}_{0}+24\;{\text{ ms}}$. These two observations indicate that solidification terminated between ${t}_{0}+24$ and ${t}_{0}+28\;{\text{ ms}}$, defining the end of Stage II. The halted increase in lattice parameter disparity in Fig. 4b indicates that no new composition is solidifying, and the maximum cooling rate corresponds to the termination of latent heat release from solidifying material. Furthermore, the corresponding temperature estimate in Fig. 4c at ${t}_{0}+24\;{\text{ ms}}$ is 991 °C, and at ${t}_{0}+28\;{\text{ ms}}$ it is 935–944 °C. These temperatures agree with the Scheil solidification curve for the MPCA, produced using ThermoCalc and displayed in Fig. 4e. The simulated curve terminates when the material is 99% solid, at 966 °C. If the simulation data are extrapolated to 100% solid, the termination temperature is 953 °C, which lies between the estimated temperatures at ${t}_{0}+24\;{\text{ ms}}$ and ${t}_{0}+28\;{\text{ ms}}$. This relative timing of the dendrite growth into a liquid pool and the advancement of the interdendritic solidification front would carry important implications into efforts to model dendritic solidification morphologies in MPCAs.

After complete solidification, the difference in lattice spacing between the dendritic and inter-dendritic regions moderates (Fig. 4b). Neglecting any difference in thermal expansion, this moderating disparity was fully attributed to interdiffusion between the regions. We define this interdiffusion period as Stage III. It lasts until ${t}_{0}+72\;{\text{ ms}}$ and an estimated temperature of 618–630 °C, after which the compositions remain largely static upon final cooling to room temperature, which we define as Stage IV. The cooling rate declines throughout Stages III and IV, as the driving force for heat transfer diminishes as the specimen temperature decreases.

## Approach for solidification and diffusion calculations

Solidification and diffusion simulations were performed using the Scheil and DICTRA modules of ThermoCalc, respectively. Both simulations employed the TCHEA4 thermodynamic database, and the DICTRA module made use of the MOBHEA2 mobility database additionally. In accordance with the finding that the diffusivity of all the elements in this MPCA is on the same order of magnitude^8^, no element was selected as a fast diffuser in the solidification simulation. For the diffusion simulation, a linear mesh of 150 points covering 400 nm was employed. To avoid simulation errors caused by discontinuous composition profiles, step-function profiles input to the diffusion simulation were subject to interfacial smoothing via the equation

2 $$C\left(x\right)= \frac{{C}_{1}+ {C}_{2}}{2}+\left(\frac{{C}_{2}- {C}_{1}}{2}\right)erf\left(\frac{x- {x}^{*}}{w}\right)$$

where *C(x)* represents the concentration of an element as a function of the linear coordinate *x*, *C*_*1*_ and *C*_*2*_ represent the concentrations on either side of the step boundary, x* represents the step boundary location, and *w* is the width of the smoothed interface. The parameter *w* was set to 5 nm for this simulation.

**Figure 6:**
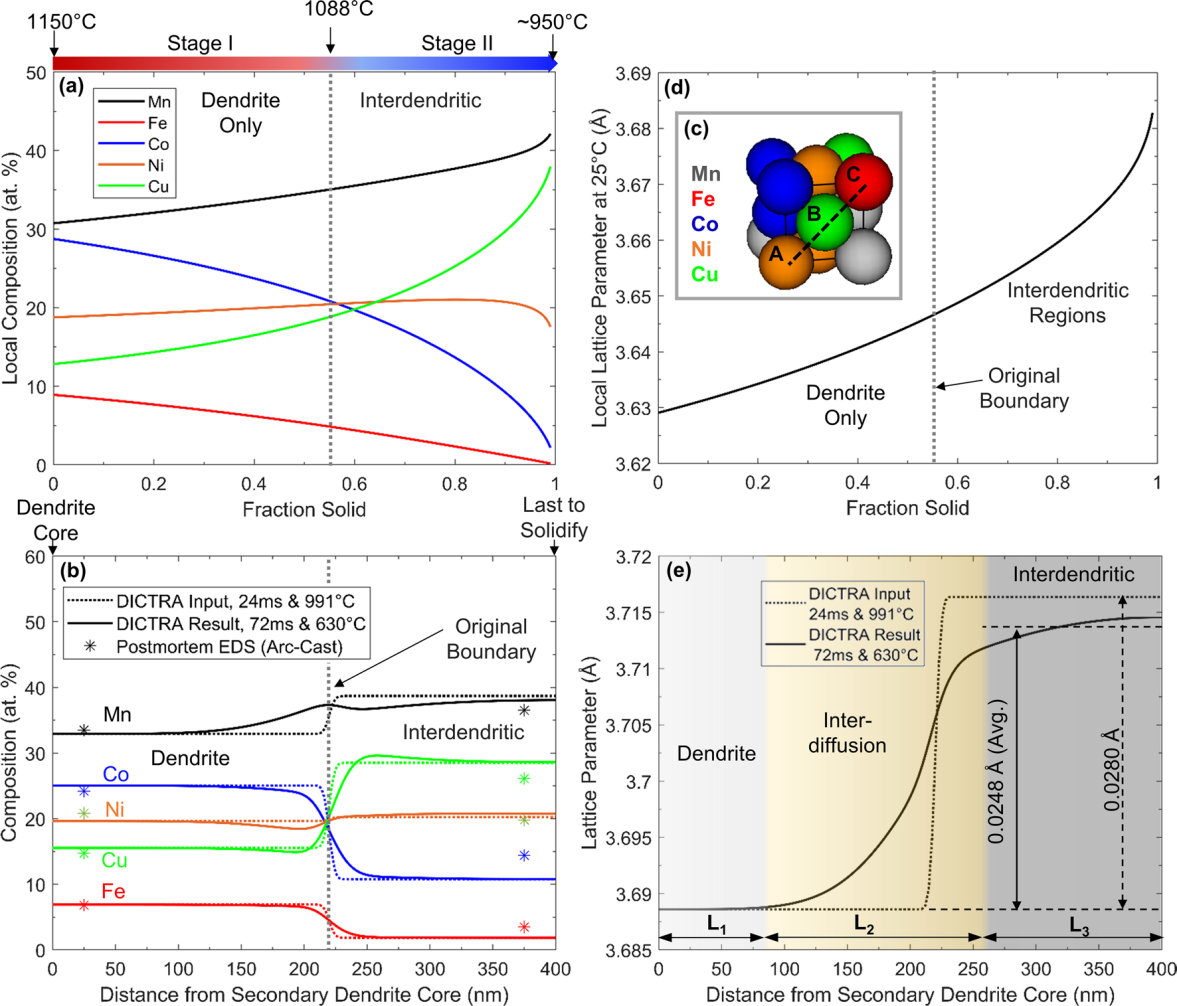
ThermoCalc simulation data for Mn_35_Fe_5_Co_20_Ni_20_Cu_20_ MPCA: (**a**) Composition profiles directly output from Scheil simulation. (**b**) Composition profiles at the termination of Stage II used as DICTRA inputs (dotted lines), and the profiles output from DICTRA at the termination of Stage III (solid lines). Final composition estimates from EDS performed on arc-cast material are displayed as stars. (**c**) Hard-sphere rendering of an example MPCA unit cell, illustrating the conversion from composition to lattice parameter (Eq. 3). (**d**) Lattice parameter profile matching the compositions in (**a**). (**e**) Lattice parameter profiles matching (**b**), including the profile at the end of Stage II (dotted line), and the profile at the end of Stage III (solid line). For simplicity, the profiles are compared at the temperature at the termination of Stage III. The dendrite (L_1_), interdiffusion (L_2_), and interdendritic (L_3_) regions identified correspond with the profile following Stage III.

The simulated transient composition evolution from the Scheil module for Stages I and II is given in Fig. 6a. These curves represent the predicted local element concentrations in the material instantaneously solidifying at a given solid fraction. As depicted, the relative concentrations of Cu and Mn increase as solidification progresses, while the concentrations of Co and Fe decrease. This behavior is qualitatively in agreement with the metallurgical analysis in Fig. 2d.

The inter-dendritic region started to solidify when the temperature reached 1088 °C, which defines the boundary between Stage I and II (Fig. 4c). As demonstrated in Fig. 4e, this corresponds to an original dendrite fraction of 0.55. The dendritic and inter-dendritic compositions that formed by 953 °C, i.e., completion of solidification at the end of Stage II, were calculated by averaging the transient composition in the simulation results from 1150 to 1088 °C for the former and from 1088 °C to 953 °C for the latter. Interfacial smoothing between these average compositions was applied as described in Eq. (2). This manipulation produced the near-step-function composition profile shown as the dotted lines in Fig. 6b, which subsequently served as the input profile for the DICTRA module, to simulate the interdiffusion in Stage III. The profile was scaled to a length of 400 nm, modeling half of a secondary dendrite arm spacing interval ($\lambda$), which was determined from Fig. 2d, and constitutes a diffusion length scale representative of the majority of the material (Fig. 2c). The location of the boundary corresponded with the overall solid fraction of 55% at the end of Stage I (Fig. 4e).

The non-isothermal DICTRA simulation was run using the temperature profiles predicted from lattice parameter evolution during Stage III, as shown in Fig. 4c. The simulation was terminated at the end of Stage III because the temperature in the majority of Stage IV was too low to be supported by the ThermoCalc’s databases, and Fig. 4b shows that compositional evolution during Stage IV is only marginal. The resulting composition profile taken at the termination of Stage III (${t}_{0}+72\;{\text{ ms}}$) is depicted as the solid lines in Fig. 6b. For validation purposes, ideally, the composition profiles should be compared to EDS measurements in the laser melted material. However, the size of the dendrites in the laser melted material was too small to accurately approximate the dendrite and inter-dendritic compositions quantitatively. Therefore, composition estimates measured from EDS on arc-cast material^8^, with dendrites approximately 10 times as large, are provided as starred markers. The composition of the dendrite cores agreed well, while the arc-cast material displays an inter-dendritic composition that is less disparate from the dendrite cores than the simulation on the laser-melt. This is reflective of a higher extent of interdiffusion in the arc-cast material, caused by a greater duration of Stage III, as arc-casting typically sees cooling rates 2–3 orders of magnitude lower than laser melting ^25^.

A hard-sphere approximation model was developed to calculate the expected room-temperature lattice parameter of a local MPCA composition, which can be directly compared to the diffraction data. Such an approach could generally be applied to a random FCC solid solution of a specific composition containing any number of elements, *n*. Under this approach, consider a single face-diagonal containing three atoms in positions A, B, and C, as shown in Fig. 6c. The atoms in the three positions can each be one of *n* types, giving n^3^ total possible arrangements for the diagonal. Assuming random ordering, the probability of a given arrangement *k* can be given as the product of the concentrations of its three constituent atoms. The FCC lattice parameter (*a*_*FCC*_) for a known local composition can then be calculated using Eq. (3):

3 $${a}_{FCC}=\left(\frac{1}{\sqrt{2}}\right)\sum_{k=1 }^{{n}^{3}}({X}_{Ak} * {X}_{Bk} * {X}_{Ck})*({r}_{Ak}+2{r}_{Bk}+{r}_{Ck})$$

The *X*_*k*_ values represent the compositional atomic fraction of the element occupying position A, B, or C for arrangement *k*, and the *r*_*k*_ values represent the atomic radii of the same elements. Using room-temperature atomic radii data from^32^, the lattice parameter profiles corresponding to the simulated composition data were calculated for the quinary MPCA (*n* = 5). The lattice parameters were then adjusted for thermal expansion, using the temperature-dependent CTE data previously discussed. The lattice parameter profiles corresponding to the simulated composition data in Fig. 6a and 6b are displayed in Fig. 6d and 6e, respectively.

**Figure 7:**
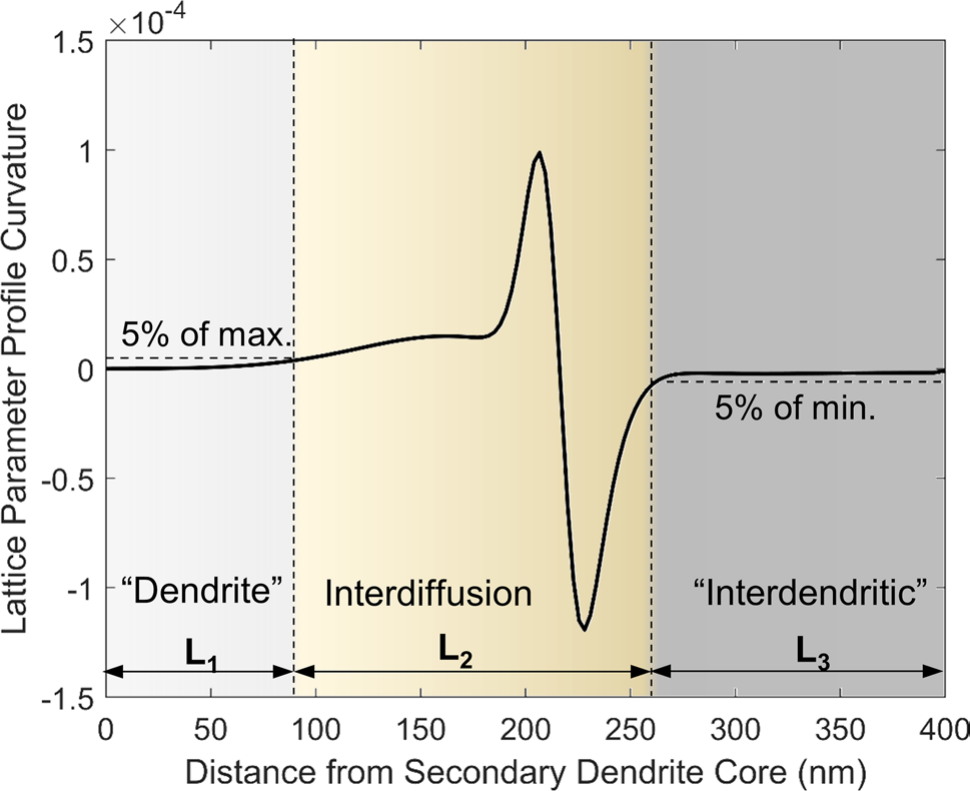
Curvature of the lattice parameter profile calculated from the DICTRA result at the end of Stage III defined by the second derivative. The definitions of the boundaries between L1, L2, and L3 are depicted.

Figure 6e shows that the lattice parameter boundary between dendrite and inter-dendritic material becomes significantly diffuse by the end of Stage III. As indicated by the shading, three regions in the final profile can be defined: (1) a zone where the dendrite lattice parameter is unaffected by interdiffusion, (2) the interdiffusion region, and (3) a zone where the profile is relatively flatter and resembles the inter-dendritic lattice parameter. In accordance with Fick’s 2nd Law of diffusion (Eq. 4)^33^, the boundaries of the interdiffusion region are defined by the locations where the second derivative (curvature) of the profile in Fig. 6e departs from zero by more than 5% of the maximum curvature (Fig. 7).

4 $$\frac{dC}{dt}=D*\frac{{d}^{2}C}{d{x}^{2}}$$

Fick’s 2nd Law is applicable because the lattice parameter profile is reflective of the composition profiles of all five elements, so the diffusion-affected region is defined as the region of nonzero curvature in lattice parameter. Taking the average of the lattice parameter profile in the inter-dendritic region, as illustrated, the predicted disparity in lattice parameter can be determined, and compared to experimental data in Fig. 4b. As depicted in Fig. 6e, the calculated disparity in the high-temperature lattice parameter between dendrite and interdendritic material is 0.0280 Å at the end of Stage II, and 0.0248 Å at the end of Stage III. These simulated values agree with the in situ experiment data in Fig. 4b to within 0.005 Å. This disparity is on the same order of magnitude as the uncertainty for much of the atomic radius data reported in^32^. Variability in fitting Pearson VII functions to a relatively coarse mesh of experimental data points (limited by the pixel-resolution of the XRD detector), as shown in Fig. 5, could have also contributed to the error.

## Dendrite fraction analysis

**Figure 8:**
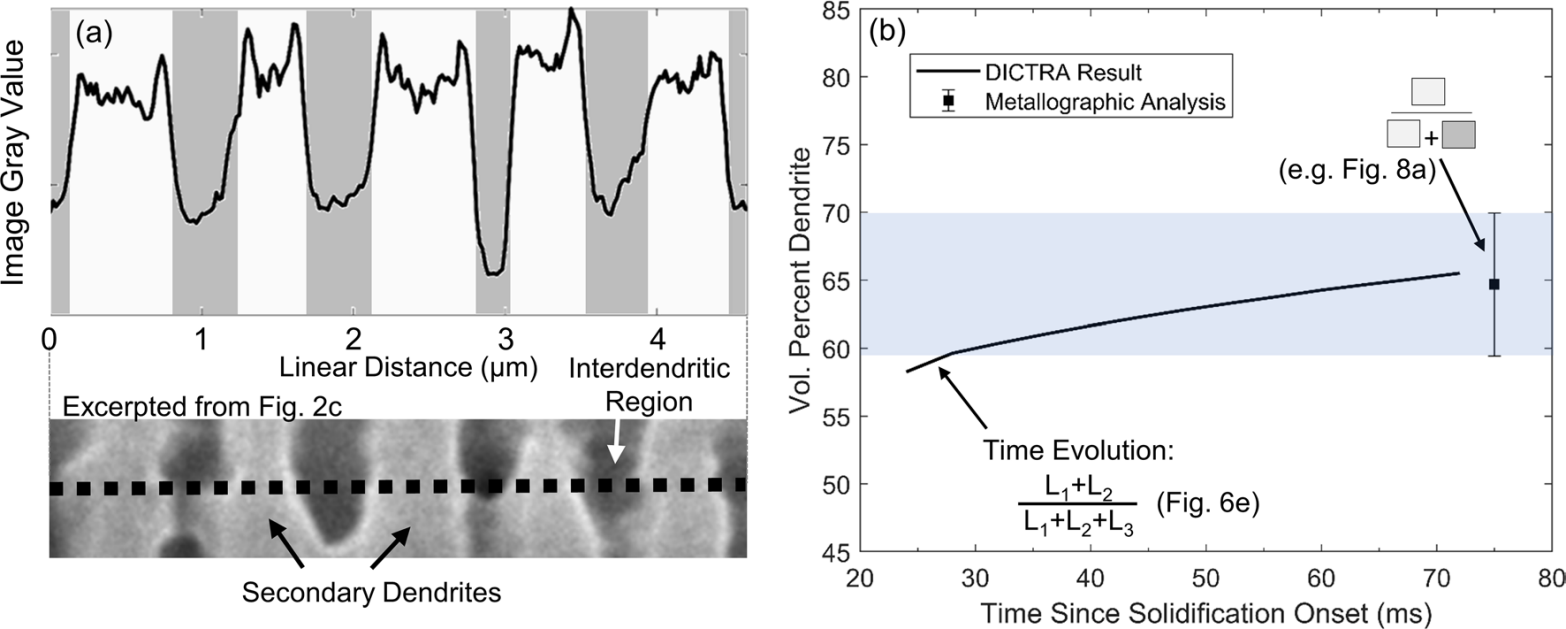
(**a**) Example gray-value profile and corresponding location on an SEM micrograph, illustrating the means of estimating dendrite fraction from postmortem metallography. (**b**) Plot comparing the dendrite fraction evolution during Stage III, calculated from DICTRA, with the dendrite fraction estimated from metallographic analysis.

Dendrite fraction was estimated through metallographic assessment. Analysis of the gray-profile was taken over six distinct series of secondary dendrite arms in the etched SEM image in Fig. 2c. An example of one such series of secondary dendrites, as well as the use of the gray-profile to delineate the dendrite and interdendritic regions, is provided in Fig. 8a. This linear profile analysis was selected over areal image thresholding to eliminate subjectivity in areal thresholding caused by gradients in the background image brightness. As depicted by the dashed lines in Fig. 8b, the six gray-profiles analyzed indicate a value of 64.7% ± 5.3% dendrite.

The dendrite fraction predicted during the course of the DICTRA simulation can be bounded below by the fraction of the full profile length composed of Line 1 (L_1_) in Fig. 6e, and bounded above by the fraction composed by the sum of L_1_ and L_2_. The upper bound and its associated equation are displayed in Fig. 8b, showing good agreement with the average value from metallography at the termination of Stage III (${t}_{0}+72\;{\text{ ms}}$), with a value of 65.5% at this time. This agreement indicates that the interdiffusion region defined in Fig. 6e displays a similar etching response to that of the dendrite core. Therefore, in metallography, the dendrite is defined as including both L_1_ and L_2_. Furthermore, the good agreement between the independent studies in Fig. 8b validates the simulation of interdiffusion behavior between dendritic and inter-dendritic regions after complete solidification using the DICTRA module.

## Conclusions

1. Mn_35_Fe_5_Co_20_Ni_20_Cu_20_ MPCA exhibited a dendritic microstructure after laser melting. Metallurgical analysis showed the dendrite is relatively rich in Fe and Co while the inter-dendritic region is rich in Mn and Cu.
2. In situ synchrotron XRD measurement during laser melting of this alloy demonstrated four distinct stages in solidification and cooling processes, including: (I) dendritic solidification of a FCC phase, (II) solidification of FCC inter-dendritic material with a slight compositional disparity, (III) solid-state interdiffusion between the dendritic and inter-dendritic regions, followed by (IV) final cooling. The timing and temperature estimates associated with each stage are identified.
3. The in situ characterization conclusively demonstrated that both Cu and Mn are rejected into liquid as Mn_35_Fe_5_Co_20_Ni_20_Cu_20_ solidifies dendritically. The relative timing of Stage I and Stage II indicate dendritic growth into a molten pool, with the interdendritic solidification front trailing by approximately 16 ms. Once interdendritic solidification started, the lattice parameter disparity between dendrite and interdendritic region escalated as Cu and Mn, elements with relatively large atomic radii, segregated into the interdendritic region.
4. Upon completion of solidification, during Stage III, interdiffusion occurred, leading to a reduced disparity in the dendritic and interdendritic lattice parameters. This finding indicates that compositional homogenization can be achieved by heat treatment in this MPCA. Only marginal interdiffusion occurred in the following Stage IV.
5. The lattice parameters calculated through the hard-sphere approximation model in combination with thermodynamic simulations and thermal expansion measurement showed good agreement with the diffraction results. Thermodynamic calculations predicted that the volume fraction of the dendrites slightly grew into the interdendritic region from 58.3% to 65.5%, which closely matched the fraction of 64.7% ± 5.3% in postmortem metallographic analysis. At each stage in the solidification process, the analysis yielded results self-consistent across different methods. This new methodology developed for solidification and diffusion analysis is therefore validated for application to complex alloys.

## Data Availability

The data that support the findings of this study are available from the corresponding authors upon reasonable request.
